# Molecular phylogeny and phylogeography of ricefishes (Teleostei: Adrianichthyidae: *Oryzias*) in Sri Lanka

**DOI:** 10.1002/ece3.9043

**Published:** 2022-06-23

**Authors:** Hiranya Sudasinghe, Tharindu Ranasinghe, Kumudu Wijesooriya, Rohan Pethiyagoda, Lukas Rüber, Madhava Meegaskumbura

**Affiliations:** ^1^ Evolutionary Ecology and Systematics Laboratory, Department of Molecular Biology and Biotechnology University of Peradeniya Peradeniya Sri Lanka; ^2^ Postgraduate Institute of Science University of Peradeniya Peradeniya Sri Lanka; ^3^ Evolutionary Ecology, Institute of Ecology and Evolution University of Bern Bern Switzerland; ^4^ Naturhistorisches Museum Bern Bern Switzerland; ^5^ Butterfly Conservation Society of Sri Lanka Malwana Sri Lanka; ^6^ Department of Zoology, Faculty of Science University of Peradeniya Peradeniya Sri Lanka; ^7^ Ichthyology Section Australian Museum Sydney New South Wales Australia; ^8^ Aquatic Ecology and Evolution, Institute of Ecology and Evolution Bern Switzerland; ^9^ Guangxi Key Laboratory for Forest Ecology and Conservation, College of Forestry Guangxi University Nanning China

**Keywords:** biodiversity hotspot, India, medaka, mtDNA, Pleistocene

## Abstract

Ricefishes of the genus *Oryzias* occur commonly in the fresh and brackish waters in coastal lowlands ranging from India across Southeast Asia and on to Japan. Among the three species of *Oryzias* recorded from peninsular India, two widespread species, *O. carnaticus* and *O. dancena*, have previously been reported from Sri Lanka based on museum specimens derived from a few scattered localities. However, members of the genus are widespread in the coastal lowlands of Sri Lanka, a continental island separated from India by the shallow Palk Strait. Although recent molecular phylogenies of Adrianichthyidae represent near‐complete taxon representation, they lack samples from Sri Lanka. Here, based on sampling at 13 locations representative of the entire geographic and climatic regions of the island's coastal lowlands, we investigate for the first time the molecular phylogenetic relationships and phylogeography of Sri Lankan *Oryzias* based on one nuclear and two mitochondrial markers. Sri Lankan *Oryzias* comprise two distinct non‐sister lineages within the javanicus species group. One of these is represented by samples exclusively from the northern parts of the island; it is recognized as *O. dancena*. This lineage is recovered as the sister group to the remaining species in the javanicus group. The second lineage represents a species that is widespread across the island's coastal lowlands. It is recovered as the sister group of *O. javanicus* and is identified as *O*. cf. *carnaticus*. Ancestral‐range estimates suggest two independent colonizations of Indian subcontinent and Sri Lanka by widespread ancestral species of *Oryzias* during two discrete temporal windows: late Miocene and Plio‐Pleistocene. No phylogeographic structure is apparent in Sri Lankan *Oryzias*, suggesting that there are no strong barriers to gene flow and dispersal along the coastal floodplains, as is the case also for other generalist freshwater fishes in the island.

## Graphical abstract

Sampling across the entire geographic and climatic regions of the island’s coastal lowlands, we investigate the molecular phylogenetic relationships and phylogeography of Sri Lankan *Oryzias*. We show that they comprise of two distinct non‐sister lineages within the javanicus species group. Ancestral‐range estimates suggest two independent colonizations of Indian subcontinent and Sri Lanka by widespread ancestral species of *Oryzias* during two discrete temporal windows: late Miocene and Plio‐Pleistocene.

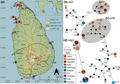

## 1 INTRODUCTION

Members of the family Adrianichthyidae, commonly called ricefishes, are small fishes inhabiting fresh and brackish waters throughout the lowlands of Southeast Asia, East Asia, and the Indian subcontinent (Parenti, 2008; Yamahira et al., 2021). The family contains two genera: *Adrianichthys*, whose four species are confined to Lake Poso in Sulawesi, and *Oryzias*, represented by 34 species (Fricke et al., 2021; Yamahira et al., 2021). More than half the known adrianichthyid species are native to Sulawesi (Mokodongan & Yamahira, 2015). Peninsular India, by comparison, has been considered to harbor only three species: *Oryzias carnaticus* (Jerdon), *O. dancena* (Hamilton), and *O. setnai* (Kulkarni) (Parenti, 2008). Among these, the former two are considered widespread: they are reported from lowland coastal habitats of eastern India, Sri Lanka, and Bangladesh (Parenti, 2008; Yamahira et al., 2021). In addition, the distribution of *O. carnaticus* and *O. dancena* extends to the Andaman Islands and Southeast Asia, respectively (Parenti, 2008; Roberts, 1998; Yamahira et al., 2021). *Oryzias setnai*, meanwhile, is confined to the lowlands of the west coast of Peninsular India, in rivers and estuaries draining into the Arabian Sea. The phylogenetic position of *O. setnai*, originally assigned to the monotypic genus *Horaichthys*, has been ambiguous. The morphology‐based phylogeny of Parenti (2008) recovered it as the sister group of the diminutive *O. uwai* Roberts from Myanmar, while recent molecular phylogenies recover it as the sister group to all other Adrianichthyidae, but with a long branch (Britz et al., 2022; Yamahira et al., 2021). The latter view is supported by *O. setnai* possessing a unique apomorphy in having the third to fifth anal‐fin rays fused into a prominent gonopodium that is more than half the length of the body. It is the only adrianichthyid species to have such a structure.

Several combinations of specific names have been variably applied to the ricefishes of Sri Lanka in the past (see Pethiyagoda, 1991). However, the taxonomic revision of the Adrianichthyidae of Parenti (2008), based on museum specimens, recognized two species (*O. carnaticus* and *O. dancena*) from the island. The Sri Lankan series examined by Parenti (2008), however, was derived from only a few scattered localities, though the genus *Oryzias* is ubiquitous in the island's coastal lowlands (Pethiyagoda, 1991; Pethiyagoda & Sudasinghe, 2021). Further, although the recent molecular phylogenies of Adrianichthyidae cited above include near‐complete taxon sampling, they lack samples from Sri Lanka.

Recent molecular phylogenetic and phylogeographic studies of freshwater fishes of Sri Lanka have revealed interesting biogeographic patterns as well as insights into their evolutionary history (Pethiyagoda & Sudasinghe, 2021). However, these studies were based principally on Cypriniformes, which are usually confined to freshwater habitats. Pethiyagoda and Sudasinghe (2021) showed that although Sri Lanka was terrestrially connected to India by the erstwhile Palk Isthmus (now submerged by the Palk Strait) for much of the Plio‐Pleistocene and until as recently as around 10,000 years ago, biotic exchange with India was mediated largely by the climate of the Isthmus. Except during brief pluvial periods, it appears to have been too arid to facilitate the dispersal of freshwater organisms between the mainland and Sri Lanka.

*Oryzias*, however, are not entirely confined to freshwaters; they occur also in brackish and estuarine environments (Pethiyagoda & Sudasinghe, 2021). In this context, a phylogeographical comparison of a widespread, saline‐tolerant species such as *Oryzias* would enhance our understanding of the biogeography of freshwater fishes in the island.

Given the lack of obvious barriers to dispersal within the island, we hypothesize a weak phylogeographic structure in the Sri Lankan ricefishes, as observed also in some of Sri Lanka's more widespread generalist cyprinid species. Further, the morphology‐based phylogeny of Parenti (2008) recovers the two species *O. carnaticus* and *O. dancena* as not closely related, whereas the molecular phylogeny of Yamahira et al. (2021) recovered them as sister species. We hypothesize a non‐sister‐group relationship between *O. carnaticus* and *O. dancena* based on their apparent morphological differences (Parenti, 2008).

To test these hypotheses, we sampled *Oryzias* from across the geographic and climatic regions of the coastal lowlands of Sri Lanka and investigate, for the first time, the molecular phylogenetic relationships, phylogeography and the ancestral‐range reconstruction of the Sri Lankan species based on a dataset derived from a combination of both mitochondrial and nuclear markers.

## 2 MATERIALS AND METHODS

### 2.1 Fieldwork

**FIGURE 1:**
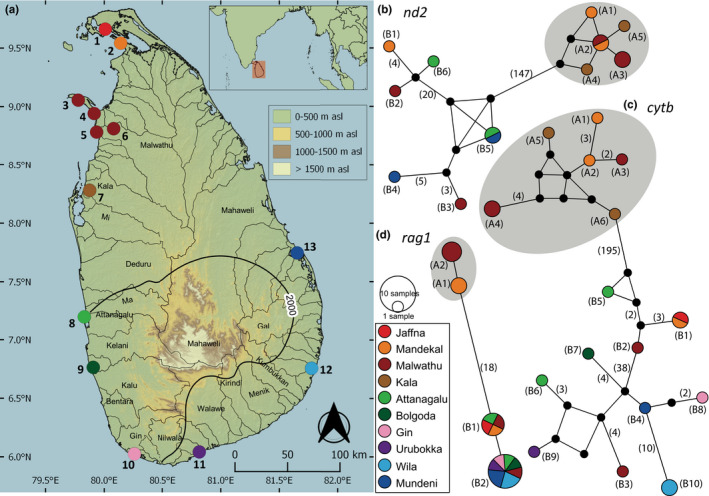
(a) Sri Lanka, with the indication of the geographical origin of samples of *Oryzias* used in the present molecular analysis. Numbers on the map represent the sampling localities listed in Table 1. Median‐joining haplotype networks for Sri Lankan *Oryzias*, based on the analysis of (b) a 711 bp fragment of the *nd2* gene, (c) a 1096 bp fragment of the *cytb* gene, and (d) a 1411 bp fragment of the *rag1* gene. The number of mutational steps >1 is shown in parentheses. The black circles are hypothetical nodes. Legend colors correspond to river basins. Haplotypes within gray circles represent *O. dancena*, while the remainder represent *O*. cf. *carnaticus*. The bold black line indicates the 2000‐mm isohyet, which encompasses the wet zone

Permission to carry out fieldwork and sampling in Sri Lanka was obtained from the Department of Wildlife Conservation (permit no. WL/3/2/59/14) and Forest Department (permit no. R&E/RES/NFSRCM/14‐16‐4) to HS and MM. The Postgraduate Institute of Science, University of Peradeniya, approved the methods of sampling and euthanasia (using tricaine methane sulfonate) at its 27th meeting held on August 4, 2017. A total of 20 specimens from 13 locations representative of the entire lowland littoral of Sri Lanka were collected (Table 1, Figure 1). Specimens were tentatively identified based on the descriptions given in Jerdon (1849: 331) and Parenti (2008). The deep‐bodied specimens with no yellow‐orange dorsal and ventral margins on the caudal fin were tentatively identified as *O. dancena*, while the shallow‐bodied specimens with yellow‐orange dorsal and ventral margins on the caudal fin were tentatively identified as *O*. cf. *carnaticus* (see Section 4).

**TABLE 1:** Details of samples of Sri Lankan *Oryzias* from which sequences were generated, with their localities, voucher references, and GenBank accession numbers

Species	Voucher	Location	GPS coordinates	*cytb*	*cytb* haplotype	*nd2*	*nd2* haplotype	*rag1*	*rag1* haplotype
*Oryzias* cf. *carnaticus*	DZ3873	Jaffna, near Jaffna Fort (1)	9.6609 N 80.0087 E	ON528961	B1	n/a	n/a	ON528981	B1
*Oryzias* cf. *carnaticus*	DZ4442	Pooneryn, Mandekal basin (2)	9.5419 N 80.1397 E	ON528962	B1	ON528999	B1	ON528982	B1
*Oryzias dancena*	DZ4444	Pooneryn, Mandekal basin (2)	9.5419 N 80.1397 E	ON528965	A2	ON529001	A2	ON528985	A1
*Oryzias dancena*	DZ4447	Pooneryn, Mandekal basin (2)	9.5419 N 80.1397 E	ON528971	A1	ON529000	A1	ON528989	A1
*Oryzias dancena*	DZ4226	Mannar, Nadukkuda (3)	9.0553 N 79.7785 E	ON528967	A4	ON528997	A3	ON528987	A2
*Oryzias dancena*	DZ4227	Mannar, Nadukkuda (3)	9.0553 N 79.7785 E	ON528968	A4	ON528998	A3	ON528988	A2
*Oryzias* cf. *carnaticus*	DZ4434	Mannar, Mannar lagoon, Malwathu basin (4)	8.9413 N 79.9139 E	ON528953	B3	ON528996	B3	ON528972	B2
*Oryzias dancena*	DZ4117	Mannar, Arippu, Malwathu basin (5)	8.7825 N 79.9346 E	ON528966	A3	ON528995	A2	ON528986	A2
*Oryzias* cf. *carnaticus*	DZ4126	Yoda ela, Malwathu basin (6)	8.8120 N 80.0800 E	ON528964	B2	ON528994	B2	ON528984	B1
*Oryzias dancena*	DZ5178	Eluwankulama, Nelum Wewa, Kala basin (7)	8.2797 N 79.8754 E	ON528969	A5	ON529002	A5	n/a	n/a
*Oryzias dancena*	DZ5179	Eluwankulama, Nelum Wewa, Kala basin (7)	8.2797 N 79.8754 E	ON528970	A6	ON529003	A4	n/a	n/a
*Oryzias* cf. *carnaticus*	DZ4453	Negombo, Attanagalu basin (8)	7.2007 N 79.8294 E	ON528955	B6	ON528990	B5	ON528974	B2
*Oryzias* cf. *carnaticus*	DZ4454	Negombo, Attanagalu basin (8)	7.2007 N 79.8294 E	ON528963	B5	ON528991	B6	ON528983	B1
*Oryzias* cf. *carnaticus*	DZ3294	Panadura, Bolgoda basin (9)	6.7637 N 79.9058 E	ON528954	B7	n/a	n/a	ON528973	B2
*Oryzias* cf. *carnaticus*	DZ4331	Galle, Unawatuna, Gin basin (10)	6.0250 N 80.2571 E	ON528956	B8	n/a	n/a	ON528975	B2
*Oryzias* cf. *carnaticus*	DZ4011	Tangalle, Urubokka basin (11)	6.0415 N 80.8167 E	ON528957	B9	n/a	n/a	ON528976	B2
*Oryzias* cf. *carnaticus*	DZ3991	Panama, Wila basin (12)	6.7556 N 81.7755 E	ON528960	B10	n/a	n/a	ON528980	B2
*Oryzias* cf. *carnaticus*	DZ3992	Panama, Wila basin (12)	6.7556 N 81.7755 E	ON528959	B10	n/a	n/a	ON528979	B2
*Oryzias* cf. *carnaticus*	DZ4504	Batticaloa, Kokkuvil, Mundeni basin (13)	7.7463 N 81.6524 E	ON528958	B4	ON528992	B4	ON528977	B2
*Oryzias* cf. *carnaticus*	DZ4505	Batticaloa, Kokkuvil, Mundeni basin (13)	7.7463 N 81.6524 E	n/a	n/a	ON528993	B5	ON528978	B2

### 2.2 DNA protocols

Gene nomenclature is based on ZFIN Zebrafish Nomenclature Conventions (https://goo.gl/MdawKQ). A total of 19 mitochondrial *cytochrome b* (*cytb*), 14 mitochondrial *NADH dehydrogenase 2* (*nd2*), and 18 nuclear *recombination activating protein 1* (*rag1*) were generated (Table 1, Figure 1). Methods of DNA extraction, PCR amplification, and PCR product purification for *cytb* and *rag1* follow Sudasinghe et al. (2020b). The primer pair ND2L (5′ GGGCCCCATACCCCAAACATGTTGG 3′) and ND2H (5’ TTAATTAAAGTGTCTGTTTTGC 3′) was used to amplify ~750 bp of the *nd2* marker (Mokodongan & Yamahira, 2015). PCR of *nd2* was carried out in 25 μl reactions, mixing 12.5 μl of GoTaq® Green Master Mix (Promega Corporation), 2 μl of template DNA (10 to 100 ng), 0.4 μl of each primer (10 μM), and 9.7 μl of deionized water. The PCR cycle of *nd2* consisted of an initial denaturation at 94°C for 1 min, followed by 35 cycles of denaturation at 94°C for 30 s, annealing at 48°C for 30 s, extension at 72°C for 1 min, and a final extension of 72°C for 10 min. PCR product purification and sequencing protocols of *nd2* were based on Sudasinghe, Ranasinghe, et al. (2018).

### 2.3 Phylogenetic analysis

ChromasPro v1.34 (Technelysium Pty Ltd) and MEGA v. 7.0 (Kumar et al., 2016) were used to verify the newly generated sequences and to prepare the consensus sequences of the 5′ and 3′ strands, respectively. The alignment of the *cytb* (1096 bp), *nd2* (711 bp) and *rag1* (1411 bp) datasets was carried out using ClustalW in MEGA, and each alignment was checked and translated to verify the absence of premature stop codons and frameshift mutations. We used the comparative dataset of *cytb*, *nd2*, and *rag1* from Yamahira et al. (2021) for our phylogenetic analysis. The third codon position of the protein coding mitochondrial genes was excluded from the phylogenetic analysis following Yamahira et al. (2021), resulting in a final 2793‐bp concatenated dataset of *cytb* + *nd2* + *rag1* for 73 taxa. Data concatenation and conversion of sequence formats was carried out using PhyloSuite v.1.2.1 (Zhang et al., 2020).

Phylogenetic inference for the 73‐taxa dataset was carried out based on a maximum‐likelihood (ML) framework using RAxML‐NG (Kozlov et al., 2019). The optimal nucleotide substitution model for the dataset was determined using ModelTest‐NG v0.1.7 (Darriba et al., 2020), providing each codon position of each gene as the starting subset, with model selection based on the Akaike information criterion (AIC). Statistical support for the nodes in the ML tree was determined by non‐parametric bootstrapping for 1000 replicates in RAxML‐NG.

The haplotype networks for *cytb*, *nd2*, and *rag1* for the populations of *Oryzias* in the island were constructed through a median‐joining network (Bandelt et al., 1999) in PopArt (Leigh & Bryant, 2015). The third codon position of the protein coding mitochondrial genes was included in reconstructing the haplotype networks.

### 2.4 Divergence‐time estimation

The divergence‐timing analysis was carried out in BEAST 2 (Bouckaert et al., 2014) on a dataset of 40 taxa. This included only the members of the order Beloniformes, reduced from the 73‐taxa dataset used in the phylogenetic analysis. We used a *cytb* substitution rate of 0.00265 substitutions per site per million years, with a normal distribution, to calibrate the *cytb* clock rate (Mokodongan & Yamahira, 2015; Takehana et al., 2005). The *nd2* and *rag1* substitution rates were estimated relative to that of *cytb*. The substitution rate for *cytb* is based on the divergence times of major lineages of Japanese *Oryzias* (Mokodongan & Yamahira, 2015; Takehana et al., 2005). A Yule pure‐birth model and a relaxed clock under lognormal distribution were used as the tree and clock prior, respectively. We carried out two independent runs consisting of 100 million generations, with the sampling interval of the Markov Chain Monte Carlo (MCMC) chain set to every 1000 generations. The convergence of the two runs was confirmed by checking if ESS > 200 for the combined run using Tracer. The first 10% generations of each run were discarded as burn‐in. The two runs were then combined using LogCombiner. A maximum clade credibility (MCC) tree was constructed using the posterior sample of trees by TreeAnnotator and visualized using FigTree v1.4.3. RAxML‐NG and the BEAST analyses were performed on UBELIX (http://www.id.unibe.ch/hpc), the HPC cluster at the University of Bern, Switzerland.

### 2.5 Ancestral‐range reconstruction

The distribution of ancestral lineages of the species of *Oryzias* present in Sri Lanka was reconstructed using the dispersal–extinction–cladogenesis (DEC) model of BioGeoBears (Matzke, 2013; Ree & Smith, 2008), as implemented in RASP 4.2 (Yu et al., 2020). The DEC analysis was run on the MCC tree obtained from the BEAST analysis. We did not impose any constraints on our model, and the maximum number of areas at ancestral ranges were specified as the maximum number of unit ranges for the most widely distributed species in each scenario. The distribution ranges of the species of ricefishes were based on Yamahira et al. (2021). We tested the distribution of ancestral lineages of Adrianichthyidae based on two different area codings: analysis 1: (A) Western Ghats, (B) Indian subcontinent (excluding Western Ghats), (C) Southeast Asia (excluding Wallacea and New Guinea), (D) East Asia, (E) Wallacea and New Guinea, and (F) Sri Lanka; analysis 2: (A) South Asia (including Western Ghats, Indian subcontinent and Sri Lanka), (B) Southeast Asia (excluding Wallacea and New Guinea), (C) East Asia, and (D) Wallacea and New Guinea. The optimal model was assessed using scores derived from the Akaike information criterion (AIC).

## 3 RESULTS

### 3.1 Molecular phylogeny

**FIGURE 2:**
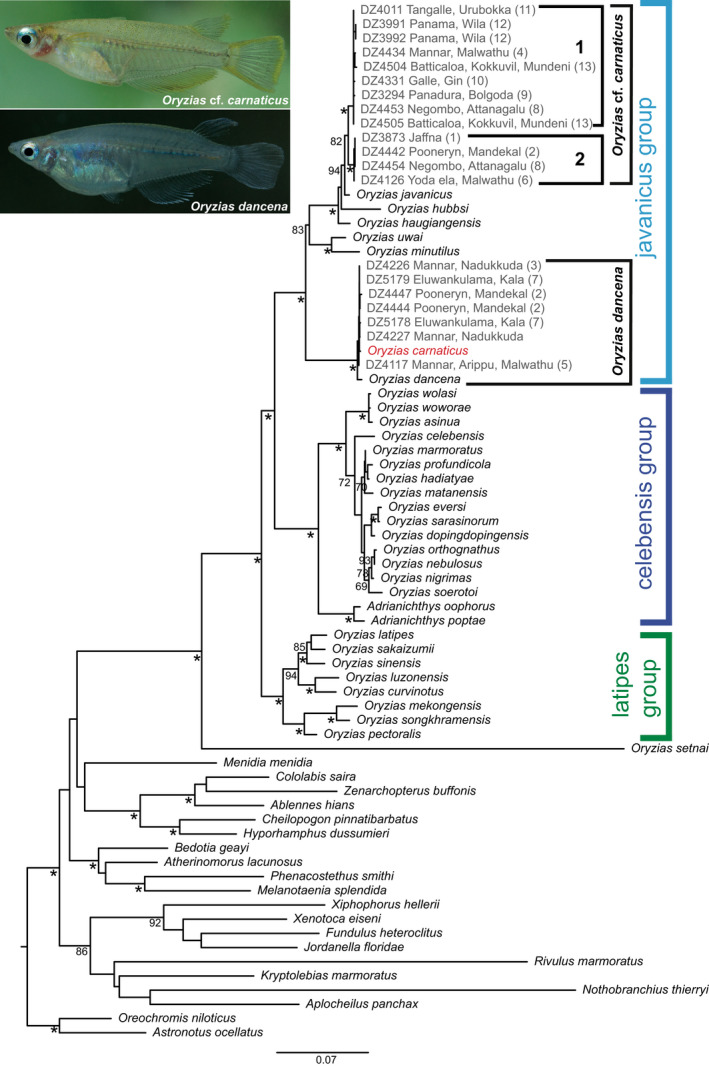
Molecular phylogenetic relationships of adrianichthyid taxa based on Maximum Likelihood inference of the concatenated *cytb* + *nd2* + *rag1* (2793 bp) dataset for 73 taxa. Asterisks (*) below nodes represent ≥95% ML non‐parametric bootstrap values. Scale bar represents the number of changes per site. Newly generated Sri Lankan sequences are in gray. Numbers in parentheses represent the sampling localities listed in Table 1. The identity of *O. carnaticus* used in Yamahira et al. (2021) is doubtful. Note the lesser body depth in *O*. cf. *carnaticus* compared with *O. dancena*, and the presence of yellow‐orange dorsal and ventral submarginal bands in the caudal fin (absent in *O. dancena*)

The ML phylogeny of the concatenated dataset of *Oryzias* recovered a topology similar to that of Yamahira et al. (2021). *Oryzias setnai* was recovered as the sister group to the remaining Adrianichthyidae, supported by a high bootstrap (>95%) value and a long branch (Figure 2). The monophyly of the three main species groups within ricefishes, the “latipes,” “celebensis,” and “javanicus” clades were well supported, with high bootstrap (>95%) values (Figure 2). The latipes group, which comprises species from East Asia, the Indochina + Sundaland and the Philippines, was recovered as the sister group to the celebensis + javanicus group with high node support (bootstrap > 95%). The celebensis group, comprised of species confined to the island of Sulawesi, and the javanicus group, comprised of species from India, Sri Lanka, and Southeast Asia, were recovered as sister groups to each other with high node support (bootstrap > 95%).

The Sri Lankan *Oryzias* represent two distinct lineages within the javanicus group, which do not show a sister‐group relationship (Figure 2). One of these, which comprises samples exclusively from the northern parts of the island, is nested with *O. dancena* and the specimen identified as *O. carnaticus* in the reference dataset of Yamahira et al. (2021) (Figure 2). This clade is recognized as *O. dancena* and is recovered as the sister group to the remaining species in the javanicus group, with high node support (bootstrap >95%). The second lineage of *Oryzias* from Sri Lanka is recovered as the sister group of *O. javanicus* (bootstrap = 94%). We tentatively recognize this clade as *O*. cf. *carnaticus* (see Section 4). *Oryzias* cf. *carnaticus* appears to be widespread across the coastal lowlands of Sri Lanka's geographic and climatic zones. Two well‐supported (bootstrap >95%) subclades of *O*. cf. *carnaticus* are recognized from the island (Figure 2). One of these, subclade 1, is widespread and represented by samples originating from the northern, southern, eastern, and western coastal lowlands, while subclade 2 is represented by samples originating only from the northern and western coastal lowlands.

### 3.2 Divergence‐time estimation

**FIGURE 3:**
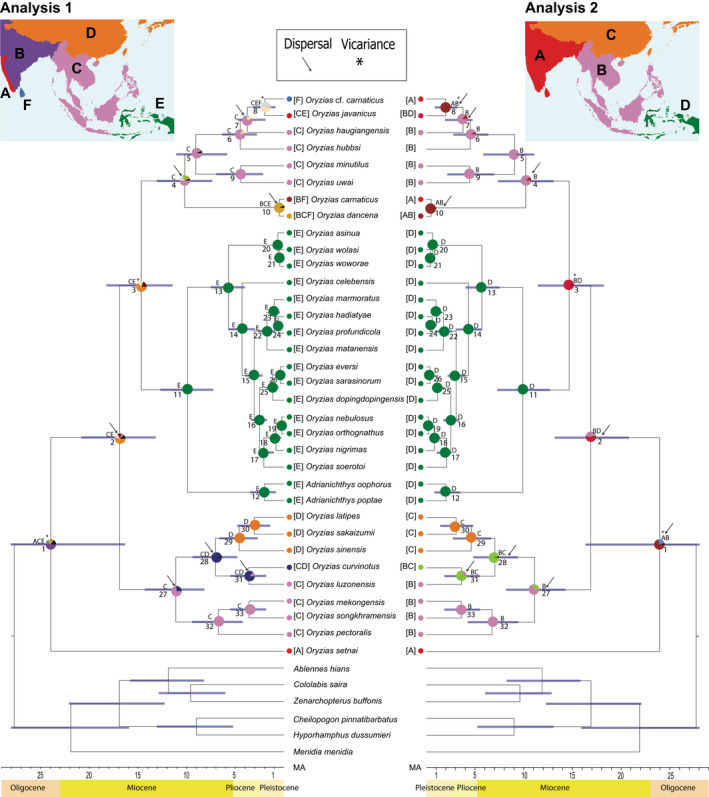
Bayesian time‐calibrated tree, based on the *cytb* substitution rate, for the concatenated dataset of *cytb* + *nd2* + *rag1* (2793 bp) for 40 taxa. Bars on the nodes indicate the 95% HPD for divergence‐time estimates. Pies at each node represent the ancestral‐range reconstructions of adrianichthyids, using the DEC model. Numbers below nodes refer to the node identifiers in Table 2

The divergence‐timing analysis for the 40‐taxa dataset using a *cytb* substitution rate in BEAST estimated the crown age of the diversification of Adrianichthyidae at 21.8 Ma (95% HPD: 16.3–28.0 Ma) in the late Oligocene to mid‐Miocene (Figure 3, Table 2). The crown ages for the latipes, celebensis, and javanicus groups were estimated as 11.1 Ma (95% HPD: 8.1–14.2 Ma), 9.9 Ma (95% HPD: 7.2–12.7 Ma), and 10.2 Ma (95% HPD: 7.3–13.0 Ma) in the late Miocene (Figure 3). Among, the two Sri Lankan species, the divergence of *O*. cf. *carnaticus* from *O*. *javanicus* was estimated to have occurred 1.9 Ma (95% HPD: 0.8–3.1 Ma), in the early Pleistocene (Figure 3).

**TABLE 2:** Comparison of the mean, 95% highest posterior density (HPD), posterior probability (PP) of the divergence‐timing analysis, and distributions suggested by the ancestral‐range reconstruction analysis^a^

Node	PP	Mean (Ma)	95% HPD (Ma)	Analysis 1	Analysis 2
1	0.7	21.8	16.3–28.0	ACE	AB
2	1	16.8	13.1–20.8	CE	BD
3	1	14.6	11.4–18.2	CE	BD
4	1	10.2	7.3–13.0	C	B
5	0.5	8.3	5.8–11.0	C	B
6	1	4.5	2.7–6.3	C	B
7	0.5	3.2	1.9–4.7	C	B
8	1	1.9	0.8–3.1	CEF	AB
9	1	4.4	2.1–6.9	C	B
10	1	0.4	0.06–1.0	BCE	AB
11	1	9.9	7.2–12.7	E	D
12	1	2.0	0.8–3.4	E	D
13	1	5.7	4.0–7.5	E	D
14	0.9	4.3	3.0–5.7	E	D
15	0.9	3.0	2.1–4.0	E	D
16	0.9	2.5	1.7–3.3	E	D
17	0.5	1.7	1.0–2.5	E	D
18	0.9	0.8	0.2–1.4	E	D
19	1	0.2	0.03–0.5	E	D
20	1	0.6	0.2–1.1	E	D
21	0.6	0.3	0.06–0.7	E	D
22	0.9	1.7	0.9–2.6	E	D
23	0.9	0.9	0.4–1.6	E	D
24	1	0.6	0.2–1.0	E	D
25	1	1.1	0.5–1.8	E	D
26	1	0.3	0.1–0.7	E	D
27	1	11.1	8.1–14.2	C	B
28	1	7.0	4.8–9.4	CD	BC
29	1	4.6	2.7–6.6	D	C
30	0.9	3.0	1.4–4.8	D	C
31	1	3.6	1.8–5.5	CD	BC
32	1	6.7	4.2–9.4	C	B
33	1	3.5	1.8–5.5	C	B

^a^ Analysis 1: (A) Western Ghats, (B) Indian subcontinent (excluding Western Ghats), (C) Southeast Asia (excluding Wallacea and New Guinea), (D) East Asia, (E) Wallacea and New Guinea, and (F) Sri Lanka; Analysis 2 (A) South Asia (including Western Ghats, Indian subcontinent, and Sri Lanka), (B) Southeast Asia (excluding Wallacea and New Guinea), (C) East Asia, and (D) Wallacea and New Guinea.

### 3.3 Ancestral‐range reconstruction

The reconstruction of ancestral ranges of ricefishes was evaluated under the DEC model, with BioGeoBEARS in RASP, under two different scenarios (Figure 3). Among the two scenarios evaluated, the best model with the lowest AIC score was obtained for analysis 2 (LnL ‐31.56, AIC 67.51) rather than analysis 1 (LnL ‐40.26, AIC 84.91). The ancestral ranges of each scenario, together with the vicariance and dispersal events, are shown in Figure 3 and Table 2.

Based on analysis 2, the most probable distribution range of the common ancestor of Adrianichthyidae was a widespread species distributed in South and Southeast Asia (Figure 3). The ancestral range of the common ancestor of the latipes, celebensis, and the javanicus groups was estimated to be widely distributed in Southeast Asia, Wallacea and New Guinea (Figure 3). The ancestral range of the latipes, celebensis, and the javanicus groups was estimated to be Southeast Asia, Wallacea and New Guinea, and Southeast Asia, respectively. For both Sri Lankan species, the ancestral range of their common ancestor was estimated to be South and Southeast Asia (Figure 3).

### 3.4 Phylogeography

Based on the median‐joining networks, the haplotypes of *O. dancena* in Sri Lanka are confined to the northern river basins such as Kala, Malwathu, and Mandekal (Figure 1). In the widespread *O*. cf. *carnaticus*, a clear phylogeographic structure within subclade 1 is not apparent, while subclade 2 appears to be confined to the northern and western coastal lowlands. In some localities, such as in Pooneryn and Negombo, samples belonging to both subclades 1 and 2 of *O*. cf. *carnaticus* are recognized (Figure 1, Table 1). In some locations in the northern region, such as from the Malwathu and Mandekal basins, both *O. dancena* and *O*. cf. *carnaticus* appear to occur in syntopy (Figure 1, Table 1).

## 4 DISCUSSION

### 4.1 Phylogenetic relationships of adrianichthyid taxa

Our molecular phylogeny corroborates the previous studies and recognizes the four main clades within Adrianichthyidae: the latipes, celebensis, and javanicus species groups of Takehana et al. (2005) and Yamahira et al. (2021) and *O. setnai*. The last‐named species was recovered as basal to the remaining Adrianichthyidae with high node support, corroborating the findings of Yamahira et al. (2021). However, Britz et al. (2022) advocate caution with regard to this phylogenetic placement of *O. setnai*, suggesting that it may be an artifact of long‐branch attraction (Felsenstein, 1978). The phylogenetic network analysis of Britz et al. (2022) for the dataset of Yamahira et al. (2021) does not recover *O. setnai* as basal to the remaining Adrianichthyidae, further supporting the phylogenetic uncertainty of this species. In contrast to the molecular phylogenetic relationship of *O. setnai*, the morphology‐based phylogeny recovers this species as the sister group of another diminutive species, *O. uwai*, from Myanmar (Parenti, 2008). A phylogenomic approach may help to resolve the higher‐level phylogenetic relationships among the taxa that constitute Adrianichthyidae (Kapli et al., 2021; Roa‐Varón et al., 2021).

### 4.2 Identity of Sri Lankan *Oryzias*

Parenti (2008), in her taxonomic revision of the Adrianichthyidae, recognized two species, *O. carnaticus* and *O. dancena*, among museum specimens collected from Sri Lanka. These two appear to be morphologically similar except that *O. dancena* is a markedly deep‐bodied species, with a body depth of 24%–34% of standard length (SL), while *O. carnaticus* has a lesser body depth of 21%–28% of SL (Parenti, 2008). She also observed the morphological similarity between *O. carnaticus* and *O. javanicus*, which too has a body depth of 24%–30% SL; these two taxa were recovered as having a sister‐group relationship in her morphology‐based phylogeny. *Oryzias carnaticus* can be distinguished from *O. javanicus* by the former having the anterior margin of the ethmoid cartilage irregular and indented anteromedially, as opposed to straight in the latter (Parenti, 2008). Further, Parenti (2008) also noted the yellow‐orange dorsal and ventral margins on the caudal fin of *O. javanicus*, which are absent in both *O. carnaticus* and *O. dancena* (Figure 2). However, this contrasts the original description of *O. carnaticus*, by Jerdon (1849: 331) who observed: “caudal edged with orange,” suggesting the same caudal fin coloration as in *O. javanicus*. The specimens identified as *O. carnaticus* from Sri Lanka by Parenti (2008) had been collected from Trincomalee (northeast coast), and Akurala (southwest coast), while the specimens she identified as *O. dancena* were derived from Trincomalee (northeast coast), Jaffna and Vaddukoddai (north coast), Colombo and Negombo (west coast), Batticaloa (east coast), and Puttalam (northwestern coast). Parenti (2008) did not state whether she examined the live coloration of *O. carnaticus* from Sri Lanka. In addition to Sri Lanka, *O. carnaticus* (type locality Vaniyambadi, Tamil Nadu, India) is also reported from Eastern India, Bangladesh, and the Andaman Islands, while *O. dancena* (type locality Kolkata, West Bengal, India) is recorded from Eastern India and Myanmar (Parenti, 2008; Roberts, 1998). Whether *O. carnaticus* occurs naturally in the Andaman Islands has been a subject of doubt (Parenti, 2008). Several recent studies report *O. javanicus* from peninsular India and the Andaman and Nicobar Islands (Angel et al., 2019; Sreeraj & Sen, 2022). However, there has been no critical evaluation of the identity of species of *Oryzias* and their distribution in this region or in Sri Lanka, even though both nominal species (*O. carnaticus* and *O. dancena*) are listed in Sri Lanka's National Red List (Goonatilake et al., 2020).

In the molecular phylogeny of Yamahira et al. (2021), *O. carnaticus* and *O. dancena* were recovered as having a mutual sister‐group relationship. The origin of their sample of *O. carnaticus* is given as Kanchipuram, on the eastern littoral of India, while that of their sample of *O. dancena* is not known (Yamahira et al., 2021). In the present study, we sampled and sequenced Sri Lankan populations of *Oryzias* and included them for the first time in a molecular phylogeny based on the reference dataset of Yamahira et al. (2021). Our molecular phylogeny too indicates two distinct lineages of *Oryzias* from Sri Lanka, both within the javanicus group. However, the two lineages were not recovered as sister species. One of these nested with the two sequences of *O. carnaticus* and *O. dancena* in Yamahira et al. (2021), while the other was recovered as the sister group of *O. javanicus* with strong node support. While our study does not address morphology, we note, based on our field observations, that the deep‐bodied samples we collected belong to the lineage that nests with *O. dancena*, while the shallow‐bodied specimens with yellow‐orange dorsal and ventral margins on the caudal fin nest as the sister group of *O. javanicus* (Figure 2). Hence, we tentatively identified this latter lineage as *O*. cf. *carnaticus*, following the original description of Jerdon (1849: 331). It is plausible that the sequence labeled as *O. carnaticus* in the reference dataset of Yamahira et al. (2021) is in fact a misidentification of *O. dancena*, while the lineage which we label as *O*. cf. *carnaticus* and recovered as the sister group of *O. javanicus* represents *O. carnaticus* sensu stricto. This is additionally credible given that Jerdon (1849) mentioned the presence of orange margins in the caudal fin in the original description of *O. carnaticus*. However, this hypothesis needs to be tested using a combination of molecular and morphological data, including examination of topotypic specimens from India, which are presently unavailable.

### 4.3 Divergence‐timing and ancestral ranges of Sri Lankan *Oryzias*

The divergence‐timing analysis of Yamahira et al. (2021), based on three fossil calibrations and the geological timing event marked by the opening of the Makassar Strait, resulted in much older age estimates for the diversification of Adrianichthyidae. For example, the study of Yamahira et al. (2021) estimated the crown ages of the diversification of Adrianichthyidae at 89 Ma (95% HPD: 73–107 Ma) and the divergence time of *O. setnai* at around 74 Ma (95% HPD: 60–88 Ma). Britz et al. (2022), however, advocate caution with regard to some of the fossil calibrations used in Yamahira et al. (2021). The divergence‐timing estimates in the present study were made primarily to understand the sequence of divergence of the Sri Lankan lineages from their most recent common ancestor. Our divergence‐timing estimates, using a *cytb* substitution rate for Adrianichthyidae, are substantially younger than those estimated by Yamahira et al. (2021). Mokodongan and Yamahira (2015) too estimated younger ages comparable to ours for the terminal nodes within the celebensis species group of the Sulawesi adrianichthyids, using only a *cytb* substitution rate. Based on our divergence‐timing and ancestral‐range estimation, two widely distributed ancestral ricefishes had colonized the Indian subcontinent and Sri Lanka twice, in two different temporal windows: one during the late Miocene and the other during the Plio‐Pleistocene (Figure 3). The first of these was the colonization by *O. dancena*, which is the sister group of the remaining members of the javanicus group, which diverged from a widespread ancestral lineage from Southeast Asia during the late Miocene and went on to colonize South Asia. The second is the more recent divergence between *O*. cf. *carnaticus* and *O. javanicus* during the Plio‐Pleistocene, from a widespread common ancestor which ranged from South to Southeast Asia. In both these cases, tectonic and climatological events in the coastal lowlands of South and Southeast Asia may explain their historical biogeography (Beck et al., 2017; Britz et al., 2022).

### 4.4 Phylogeography and genetic diversity of Sri Lankan adrianichthyids

Previous studies exploring comparative phylogeographic patterns and genetic structure in Sri Lankan freshwater fishes focused primarily on Cypriniformes, which are obligatorily confined to freshwater habitats (Sudasinghe, Dahanukar, et al., 2021; Sudasinghe et al., 2020a, 2020b; Sudasinghe, Herath, et al., 2018; Sudasinghe, Pethiyagoda, Raghavan, et al., 2020; Sudasinghe, Pethiyagoda, Ranasinghe, et al., 2020; Sudasinghe, Raghavan, et al., 2021; Sudasinghe, Ranasinghe, et al., 2021). In contrast, Sri Lankan adrianichthyids offer us, for the first time, an opportunity to explore the phylogeography of a widespread, saline‐tolerant species. As hypothesized, we did not find any strong phylogeographic structure in the two species of Sri Lankan adrianichthyids. The pattern observed here is similar to that observed also in widespread generalist cyprinids such as in *Dawkinsia filamentosa* (Valenciennes), *Devario malabaricus* (Jerdon), *Rasbora dandia* (Valenciennes) and the snakehead, *Channa kelaartii* (Günther) in Sri Lanka (Sudasinghe, Pethiyagoda, Ranasinghe, et al., 2020; Sudasinghe, Pethiyagoda, Meegaskumbura, et al., 2020; Sudasinghe et al., 2020b; Sudasinghe, Raghavan, et al., 2021). As in those species, it appears that gene flow in the island's adrianichthyids occurs freely along the lowland coastal floodplain, across which there are no physical barriers to dispersal. However, within the widespread *O*. cf. *carnaticus*, we observe two well‐supported subclades. One of these, subclade 1, is a widespread lineage represented by samples from throughout the island's coastal lowlands, while subclade 2 is confined to the northern and western coastal lowlands. In some sampled localities in the northern and western coastal lowlands, representatives of both subclades occur in syntopy. The syntopic occurrence of genetically distinct mitochondrial lineages has been observed also in the Sri Lankan cyprinids *Garra ceylonensis* Bleeker and *Pethia nigrofasciata* (Günther); it may suggest that each of these populations represents historically separate evolutionary lineages (Sudasinghe, Dahanukar, et al., 2021; Sudasinghe, Ranasinghe, et al., 2021). In contrast to *O*. cf. *carnaticus*, our samples of *O. dancena* derived only from the northern coastal regions of the island despite Parenti (2008) having recorded the latter species from several localities in the east and west coast as well. It is interesting to note that at some localities in the northern coast, we recorded both species in syntopy.

Pethiyagoda and Sudasinghe (2021) noted that “owing to aridity, the Palk Isthmus appears to have served more as a filter of—than as a conduit for—biotic dispersal as the Plio‐Pleistocene advanced.” A dearth of samples from southern India precluded us from assessing whether this holds true also for saline‐tolerant fishes such as *Oryzias*.

Our results suggest that the systematics of *Oryzias* in Sri Lanka is more complex than was previously thought. Finer sampling throughout the coastal lowlands of the island accompanied by taxonomic revision based on morphological and genetic analyses will help construct a more complete picture of the identity and distribution of the adrianichthyids of Sri Lanka and South Asia.

## AUTHOR CONTRIBUTIONS

**Hiranya Sudasinghe:** Conceptualization (equal); data curation (lead); formal analysis (lead); investigation (lead); methodology (equal); validation (equal); visualization (equal); writing – original draft (equal); writing – review and editing (equal). **Tharindu Ranasinghe:** Data curation (equal); investigation (equal); validation (equal); writing – review and editing (equal). **Kumudu Wijesooriya:** Data curation (equal); investigation (equal); validation (equal); writing – review and editing (equal). **Rohan Pethiyagoda:** Conceptualization (equal); funding acquisition (equal); project administration (equal); resources (equal); supervision (equal); writing – original draft (equal); writing – review and editing (equal). **Lukas Ruber:** Conceptualization (equal); funding acquisition (equal); resources (equal); supervision (equal); writing – original draft (equal); writing – review and editing (equal). **Madhava Meegaskumbura:** Conceptualization (equal); funding acquisition (equal); methodology (equal); resources (equal); supervision (equal); writing – review and editing (equal).

## CONFLICT OF INTEREST

The authors declare no competing interests.

## Data Availability

All data generated or analyzed during this study are included in this published article and available in the NCBI database (https://www.ncbi.nlm.nih.gov/). The newly generated *cytb*, *nd2*, and *rag1* sequences in this study are deposited in GenBank under accession numbers ON528953–ON528971, ON528990–ON529003, and ON528972–ON528989, respectively.

## APPENDIX 1 Nucleotide substitution models and the partitions used in the phylogenetic analyses

**Table:**

Analysis	Gene	Number of sequences	Partition	Model
Maximum‐likelihood inference: RAxML‐NG	*cytb* + *nd2* + *rag1* (2793 bp)	73	*cytb* 1st	TVMe+I + G4
			*cytb* 2nd	TPM2 + F + I + G4
			*nd2* 1st	TVM + F + I + G4
			*nd2* 2nd	TVM + F + G4
			*rag1* 1st	TIM2 + F + I + G4
			*rag1* 2nd	K3P + I + G4
			*rag1* 3rd	TIM2e + G4
Starting tree for divergence‐timing analysis: BEAST	*cytb* + *nd2* + *rag1* (2793 bp)	40	*cytb*	HKY + I + G
			*nd2*	TRN + I + G
			*rag1*	K80 + G

## References

[ece39043-bib-0001] Angel, J. R. J. , Vinay, T. N. , Raghavan, R. , Thomas, D. , Avunje, S. , Aravind, R. , Shekhar, M. S. , & Vijayan, K. K. (2019). First record of the Javanese ricefish, *Oryzias javanicus* (Bleeker, 1854) (Beloniformes: Adrianichthyidae) in the natural waters of India. Journal of Applied Ichthyology, 35(4), 1034–1038. 10.1111/jai.13933

[ece39043-bib-0002] Bandelt, H. J. , Forster, P. , & Röhl, A. (1999). Median‐joining networks for inferring intraspecific phylogenies. Molecular Biology and Evolution, 16(1), 37–48. 10.1093/oxfordjournals.molbev.a026036 10331250

[ece39043-bib-0003] Beck, S. V. , Carvalho, G. R. , Barlow, A. , Rüber, L. , Hui Tan, H. , Nugroho, E. , Wowor, D. , Mohd Nor, S. A. , Herder, F. , Muchlisin, Z. A. , & de Bruyn, M. (2017). Plio‐Pleistocene phylogeography of the Southeast Asian Blue Panchax killifish, *Aplocheilus panchax* . PLoS One, 12(7), e0179557. 10.1371/journal.pone.0179557 28742862 PMC5526567

[ece39043-bib-0004] Bouckaert, R. , Heled, J. , Kühnert, D. , Vaughan, T. , Wu, C.‐H. , Xie, D. , Suchard, M. A. , Rambaut, A. , & Drummond, A. J. (2014). BEAST 2: A software platform for Bayesian evolutionary analysis. PLoS Computational Biology, 10(4), e1003537. 10.1371/journal.pcbi.1003537 24722319 PMC3985171

[ece39043-bib-0005] Britz, R. , Parenti, L. R. , & Rüber, L. (2022). Earth and life evolve together – A comment on Yamahira et al. Biology Letters, 18, 20210568. 10.1098/rsbl.2021.0568 35350877 PMC8965416

[ece39043-bib-0006] Darriba, D. , Posada, D. , Kozlov, A. M. , Stamatakis, A. , Morel, B. , & Flouri, T. (2020). ModelTest‐NG: A new and scalable tool for the selection of DNA and Protein evolutionary models. Molecular Biology and Evolution, 37(1), 291–294. 10.1093/molbev/msz189 31432070 PMC6984357

[ece39043-bib-0007] Felsenstein, J. (1978). Cases in which parsimony or compatibility methods will be positively misleading. Systematic Biology, 27(4), 401–410. 10.1093/sysbio/27.4.401

[ece39043-bib-0008] Fricke, R. , Eschmeyer, W. N. , & Van der Laan, R. (2021). Eschmeyer's catalog of fishes: Genera, species, references . http://researcharchive.calacademy.org/research/ichthyology/catalog/fishcatmain.asp

[ece39043-bib-0009] Goonatilake, S. A. , Fernando, M. , Kotagama, O. , Perera, N. , VIdanage, S. , Weerakoon, D. , Adam, A. , & Maiz‐Tome, L. (2020). The National Red List of Sri Lanka: Assessment of the Threat Status of the Freshwater Fishes of Sri Lanka 2020. IUCN, International Union for Conservation of Nature, Sri Lanka, the Biodiversity Secretariat, Ministry of Environment and Wildlife Resources.

[ece39043-bib-0010] Jerdon, T. C. (1849). On the fresh water fishes of southern India. Madras Journal of Literature and Science, 15, 302–346.

[ece39043-bib-0011] Kapli, P. , Flouri, T. , & Telford, M. J. (2021). Systematic errors in phylogenetic trees. Current Biology, 31(2), R59–R64. 10.1016/j.cub.2020.11.043 33497629

[ece39043-bib-0012] Kozlov, A. M. , Darriba, D. , Flouri, T. , Morel, B. , & Stamatakis, A. (2019). RAxML‐NG: A fast, scalable and user‐friendly tool for maximum likelihood phylogenetic inference. Bioinformatics, 35(21), 4453–4455. 10.1093/bioinformatics/btz305 31070718 PMC6821337

[ece39043-bib-0013] Kumar, S. , Stecher, G. , & Tamura, K. (2016). MEGA7: Molecular Evolutionary Genetics Analysis version 7.0 for bigger datasets. Molecular Biology and Evolution, 33(7), 1870–1874. 10.1093/molbev/msw054 27004904 PMC8210823

[ece39043-bib-0014] Leigh, J. W. , & Bryant, D. (2015). POPART: Full‐feature software for haplotype network construction. Methods in Ecology and Evolution, 6(9), 1110–1116. 10.1111/2041-210X.12410

[ece39043-bib-0015] Matzke, N. J. (2013). Probabilistic historical biogeography: New models for founder‐event speciation, imperfect detection, and fossils allow improved accuracy and model‐testing. Frontiers in Biogeography, 5, 242–248. 10.21425/F55419694

[ece39043-bib-0016] Mokodongan, D. F. , & Yamahira, K. (2015). Origin and intra‐island diversification of Sulawesi endemic Adrianichthyidae. Molecular Phylogenetics and Evolution, 93, 150–160. 10.1016/j.ympev.2015.07.024 26256644

[ece39043-bib-0017] Parenti, L. R. (2008). A phylogenetic analysis and taxonomic revision of ricefishes, *Oryzias* and relatives (Beloniformes, Adrianichthyidae). Zoological Journal of the Linnean Society, 154(3), 494–610. 10.1111/j.1096-3642.2008.00417.x

[ece39043-bib-0018] Pethiyagoda, R. (1991). Freshwater fishes of Sri Lanka. Wildlife Heritage Trust.

[ece39043-bib-0019] Pethiyagoda, R. , & Sudasinghe, H. (2021). The ecology and biogeography of Sri Lanka: A context for freshwater fishes. WHT Publications (Private) Limited.

[ece39043-bib-0020] Ree, R. H. , & Smith, S. A. (2008). Maximum likelihood inference of geographic range evolution by dispersal, local extinction, and cladogenesis. Systematic Biology, 57(1), 4–14. 10.1080/10635150701883881 18253896

[ece39043-bib-0021] Roa‐Varón, A. , Dikow, R. B. , Carnevale, G. , Tornabene, L. , Baldwin, C. C. , Li, C. , & Hilton, E. J. (2021). Confronting sources of systematic error to resolve historically contentious relationships: A case study using gadiform fishes (Teleostei, Paracanthopterygii, Gadiformes). Systematic Biology, 70(4), 739–755. 10.1093/sysbio/syaa095 33346841 PMC8561434

[ece39043-bib-0022] Roberts, T. R. (1998). Systematic observations on tropical Asian medakas or ricefishes of the genus *Oryzias*, with descriptions of four new species. Ichthyological Research, 45(3), 213–224. 10.1007/BF02673919

[ece39043-bib-0023] Sreeraj, C. R. , & Sen, A. (2022). Javanese Rice Fish (*Oryzias javanicus*)—First Record from Andaman and Nicobar Islands. Asian Basic and Applied Research Journal, 5(3), ABAARJ.881.

[ece39043-bib-0024] Sudasinghe, H. , Dahanukar, N. , Raghavan, R. , Senavirathna, T. , Shewale, D. J. , Paingankar, M. S. , Amarasinghe, A. , Pethiyagoda, R. , Rüber, L. , & Meegaskumbura, M. (2021). Island colonization by a ‘rheophilic’ fish: The phylogeography of *Garra ceylonensis* (Teleostei: Cyprinidae) in Sri Lanka. Biological Journal of the Linnean Society, 132(4), 872–893. 10.1093/biolinnean/blaa221

[ece39043-bib-0025] Sudasinghe, H. , Herath, J. , Pethiyagoda, R. , & Meegaskumbura, M. (2018). Undocumented translocations spawn taxonomic inflation in Sri Lankan fire rasboras (Actinopterygii, Cyprinidae). PeerJ, 6, e6084. 10.7717/peerj.6084 30595978 PMC6304270

[ece39043-bib-0026] Sudasinghe, H. , Pethiyagoda, R. , & Meegaskumbura, M. (2020a). A molecular phylogeny of the genus *Laubuka* (Teleostei: Cyprinidae) in Sri Lanka reveals multiple origins and a cryptic species. Systematics and Biodiversity, 18(6), 592–613. 10.1080/14772000.2020.1771468

[ece39043-bib-0027] Sudasinghe, H. , Pethiyagoda, R. , & Meegaskumbura, M. (2020b). Evolution of Sri Lanka's Giant Danios (Teleostei: Cyprinidae: *Devario*): Teasing apart species in a recent diversification. Molecular Phylogenetics and Evolution, 149, 106853. 10.1016/j.ympev.2020.106853 32417495

[ece39043-bib-0028] Sudasinghe, H. , Pethiyagoda, R. , Meegaskumbura, M. , Maduwage, K. , & Britz, R. (2020). Channa kelaartii, a valid species of dwarf snakehead from Sri Lanka and southern peninsular India (Teleostei: Channidae). Vertebrate Zoology, 70(2), 157–170. 10.26049/VZ70-2-2020-05

[ece39043-bib-0029] Sudasinghe, H. , Pethiyagoda, R. , Raghavan, R. , Dahanukar, N. , Rüber, L. , & Meegaskumbura, M. (2020). Diversity, phylogeny and biogeography of *Systomus* (Teleostei, Cyprinidae) in Sri Lanka. Zoologica Scripta, 49, 710–731. 10.1111/zsc.12445

[ece39043-bib-0030] Sudasinghe, H. , Pethiyagoda, R. , Ranasinghe, R. H. T. , Raghavan, R. , Dahanukar, N. , & Meegaskumbura, M. (2020). A molecular phylogeny of the freshwater‐fish genus *Rasbora* (Teleostei: Cyprinidae) in Sri Lanka reveals a remarkable diversification‐and a cryptic species. Journal of Zoological Systematics and Evolutionary Research, 58, 1076–1110. 10.1111/jzs.12395

[ece39043-bib-0031] Sudasinghe, H. , Raghavan, R. , Dahanukar, N. , Pethiyagoda, R. , Rüber, L. , & Meegaskumbura, M. (2021). Diversification and biogeography of *Dawkinsia* (Teleostei: Cyprinidae) in the Western Ghats‐Sri Lanka Biodiversity Hotspot. Organisms Diversity & Evolution, 21, 795–820. 10.1007/s13127-021-00515-x

[ece39043-bib-0032] Sudasinghe, H. , Ranasinghe, R. H. T. , Goonatilake, S. A. , & Meegaskumbura, M. (2018). A review of the genus *Labeo* (Teleostei: Cyprinidae) in Sri Lanka. Zootaxa, 4486(3), 201–235. 10.11646/zootaxa.4486.3.1 30313744

[ece39043-bib-0033] Sudasinghe, H. , Ranasinghe, T. , Herath, J. , Wijesooriya, K. , Pethiyagoda, R. , Rüber, L. , & Meegaskumbura, M. (2021). Molecular phylogeny and phylogeography of the freshwater‐fish genus *Pethia* (Teleostei: Cyprinidae) in Sri Lanka. BMC Ecology and Evolution, 21, 203. 10.1186/s12862-021-01923-5 34758736 PMC8582130

[ece39043-bib-0034] Takehana, Y. , Naruse, K. , & Sakaizumi, M. (2005). Molecular phylogeny of the medaka fishes genus *Oryzias* (Beloniformes: Adrianichthyidae) based on nuclear and mitochondrial DNA sequences. Molecular Phylogenetics and Evolution, 36(2), 417–428. 10.1016/j.ympev.2005.01.016 15955519

[ece39043-bib-0035] Yamahira, K. , Ansai, S. , Kakioka, R. , Yaguchi, H. , Kon, T. , Montenegro, J. , Kobayashi, H. , Fujimoto, S. , Kimura, R. , Takehana, Y. , Setiamarga, D. H. E. , Takami, Y. , Tanaka, R. , Maeda, K. , Tran, H. D. , Koizumi, N. , Morioka, S. , Bounsong, V. , Watanabe, K. , … Kitano, J. (2021). Mesozoic origin and ‘out‐of‐India’ radiation of ricefishes (Adrianichthyidae). Biology Letters, 17(8), 20210212. 10.1098/rsbl.2021.0212 34343438 PMC8331229

[ece39043-bib-0036] Yu, Y. , Blair, C. , & He, X. (2020). RASP 4: Ancestral state reconstruction tool for multiple genes and characters. Molecular Biology and Evolution, 37(2), 604–606. 10.1093/molbev/msz257 31670774

[ece39043-bib-0037] Zhang, D. , Gao, F. , Jakovlić, I. , Zou, H. , Zhang, J. , Li, W. X. , & Wang, G. T. (2020). PhyloSuite: An integrated and scalable desktop platform for streamlined molecular sequence data management and evolutionary phylogenetics studies. Molecular Ecology Resources, 20(1), 348–355. 10.1111/1755-0998.13096 31599058

